# A Costing Analysis of a Nurse- and Peer-Led Mobile Model of Hepatitis C Care Adjacent to the Community Corrections Setting

**DOI:** 10.1093/ofid/ofag135

**Published:** 2026-03-17

**Authors:** Samara Griffin, Farah Houdroge, Nick Scott, Bridget Reid, Kelly Maynard, Alex Pappas, Anne Craigie, Mark Belzer, Jane Dicka, Jacinta A Holmes, Alexander J Thompson, Mark Stoove, Rebecca Winter

**Affiliations:** School of Public Health and Preventive Medicine, Monash University, Melbourne, Victoria, Australia; Disease Elimination, Burnet Institute, Melbourne, Victoria, Australia; Department of Gastroenterology, St Vincent's Hospital, Melbourne, Victoria, Australia; Disease Elimination, Burnet Institute, Melbourne, Victoria, Australia; School of Public Health and Preventive Medicine, Monash University, Melbourne, Victoria, Australia; Disease Elimination, Burnet Institute, Melbourne, Victoria, Australia; Department of Gastroenterology, St Vincent's Hospital, Melbourne, Victoria, Australia; Disease Elimination, Burnet Institute, Melbourne, Victoria, Australia; Department of Gastroenterology, St Vincent's Hospital, Melbourne, Victoria, Australia; Department of Gastroenterology, St Vincent's Hospital, Melbourne, Victoria, Australia; Harm Reduction Victoria, Melbourne, Victoria, Australia; Harm Reduction Victoria, Melbourne, Victoria, Australia; Department of Gastroenterology, St Vincent's Hospital, Melbourne, Victoria, Australia; Department of Medicine, University of Melbourne, Melbourne, Victoria, Australia; Department of Gastroenterology, St Vincent's Hospital, Melbourne, Victoria, Australia; Department of Medicine, University of Melbourne, Melbourne, Victoria, Australia; School of Public Health and Preventive Medicine, Monash University, Melbourne, Victoria, Australia; Disease Elimination, Burnet Institute, Melbourne, Victoria, Australia; Australian Research Centre in Sex, Health and Society, La Trobe University, Melbourne, Victoria, Australia; School of Public Health and Preventive Medicine, Monash University, Melbourne, Victoria, Australia; Disease Elimination, Burnet Institute, Melbourne, Victoria, Australia; Department of Gastroenterology, St Vincent's Hospital, Melbourne, Victoria, Australia

**Keywords:** correctional setting, cost, hepatitis C virus, mobile services, point-of-care testing

## Abstract

**Background:**

Prison-based hepatitis C models of care treat significant numbers of people in Australia but exclude most of the criminal justice system, individuals serving their sentence in the community, on community corrections orders. We estimate the cost of a nurse- and peer-led model of hepatitis C care, offering hepatitis C virus (HCV) point-of-care testing and treatment initiation, adjacent to the community corrections setting in Melbourne, Australia.

**Methods:**

Data from September 2023 to March 2025 for the C No More program were incorporated. All costs were estimated from a service provider perspective, including clinic infrastructure, staff wages, testing, incentive, and pharmacy costs. Treatment and pretreatment pathology costs were excluded as these are covered by the Australian government. Total program cost, mean cost per individual initiated on treatment, and mean cost per individual that achieved sustained virological response were calculated.

**Results:**

The total cost of the C No More service between September 2023 and March 2025 was A$312 166.85 (costs are presented in 2024 Australian dollars). Among 832 participants, 592 were HCV antibody tested, 275 were HCV RNA tested, 61 were HCV RNA positive, 58 initiated treatment, and 41 achieved sustained virological response. The cost per participant initiating treatment was A$5382.19, and the cost per participant cured was A$7613.83. HCV RNA–negative participants (n = 771 [93%]) contributed to 84% of total program costs (A$263 490.72).

**Conclusions:**

Providing effective hepatitis C care in the community requires a low-threshold, supportive model of care with intensive follow-up and retention in care strategies. Service costs reflect increased case finding challenges, in the context of declining community HCV RNA prevalence.

## Teaser

A low-threshold, nurse- and peer-led model of outreach care adjacent to the community corrections setting tested many at-riskpeople for hepatitis C. The service cost, A$5382.19 per person initiating treatment, is reflective of the supportive model and increasing case-finding costs.

## Graphical Abstract

**Graphical Abstract:**
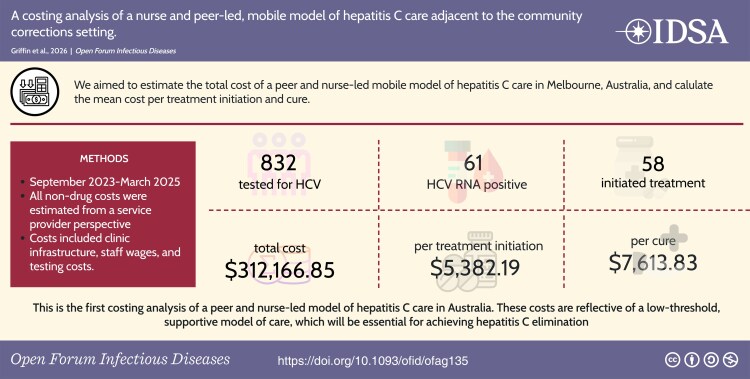
Tidbit Graphical Abstract: https://tidbitapp.io/tidbits/a-costing-analysis-of-a-nurse-and-peer-led-mobile-model-of-hepatitis-c-care-adjacent-to-the-community-corrections-setting?utm_campaign=tidbitlinkshare&amp;utm_source=ITP

Accessible and adapted models of hepatitis C care that address barriers to accessing testing and treatment through traditional health service models will be crucial for achieving hepatitis C elimination [1]. As countries progress toward World Health Organization hepatitis C elimination goals, the costs of finding and treating those who remain to be treated will increase [1, 2]. Models of care that are both accessible and cost effective will be important for achieving and maintaining elimination [3].

Integration of hepatitis C care into services used by people who inject drugs is recommended by the World Health Organization as a way of providing accessible care [4]. The criminal justice system is a priority setting for people who use drugs and are at risk of hepatitis C; however, hepatitis programs in justice systems have typically been restricted to care provided in prison settings [5]. In Australia, prison-based hepatitis programs treat large numbers of people [6] but exclude the majority of those in the criminal justice system who serve their sentences in the community via community corrections orders [6, 7]. The community corrections (probation and parole) and incarcerated populations share similar risk factors for hepatitis C, with similar levels of injecting drug use (IDU) [8, 9].

While the primary focus of community corrections is administrative, the justice system is increasingly recognized as an opportunity to provide social and healthcare services to a group of people who may be marginalized from mainstream services [8, 10–12]. Community corrections orders typically require regular reporting to a designated corrections office, which provides a potential touchpoint to provide hepatitis C care and a transitional care opportunity for individuals who may fall in the gap between prison and the community hepatitis C care.

Globally, models of care offering hepatitis C testing and treatment based in community corrections have been piloted, with varying degrees of clinical effectiveness [13–15], but their costs have not been reported. In the current study, we aimed to estimate the total cost from a service providers perspective for a nurse- and peer-led mobile model of hepatitis C care in Melbourne, Australia [16], and we calculated the mean cost per HCV RNA–positive diagnosis, cost per treatment initiation and cost per cure. We also explored the distribution of costs across individuals and factors that may influence mean costs associated with HCV RNA–positive individuals.

## METHODS

### Study Design and Setting

The C No More model of care was implemented as a pilot feasibility study that aimed to provide hepatitis C testing and treatment to individuals on community corrections orders and members of the wider community. Details of the C No More study have been published elsewhere [16]. Briefly, C No More was a nurse- and peer-led mobile model of hepatitis C care, offering point-of-care hepatitis C testing and rapid direct-acting antiviral (DAA) treatment initiation adjacent to community corrections offices in Melbourne, Victoria, in Australia. The service operated inside a clinically equipped van parked adjacent to 4 metropolitan community corrections offices, and it was staffed by a hepatology clinical nurse consultant and 2 harm reduction peer workers.

Individuals were screened for hepatitis C virus (HCV) antibody using INSTI or Abbott Bioline HCV antibody tests, depending on availability for ordering. If testing antibody positive (or self-reported antibody positive), individuals were tested for HCV RNA using the Cepheid GeneXpert HCV RNA test. HCV RNA–positive individuals underwent venipuncture for pretreatment blood-based investigations, including liver function and blood-borne virus testing. DAA treatment was prescribed in consultation with a nurse practitioner or gastroenterologist and dispensed through a local pharmacy or through the van via the affiliated hospital pharmacy, at the individual's choosing. Hepatitis C RNA–positive individuals were provided with person-centered, flexible support by the nurse and peer workers throughout treatment, including regular telephone check-ins and follow-up, flexible dispensing, and linkage to other health and social services. This study was registered with the Australian New Zealand Clinical Trials Registry (ACTRN12623001043628).

### Data Source

The study commenced in September 2023, and data for this analysis used clinical and operational data to the end of March 2025, as well as publicly available administrative data. Clinical data, including demographic, testing, treatment, and follow-up data, were recorded on a secured REDCap (Research Electronic Data Capture) database hosted by Burnet Institute. Operational data included staff salaries and the costs associated with the van, fuel, testing resources, and consumables. Publicly available data included the cost of HCV antibody INSTI or Abbott Bioline tests and HCV RNA GeneXpert test cartridges, which were provided to the program by the Australian HCV Point-of-Care Testing Program [17].

### Costing Analysis

The costing analysis adopted a service provider perspective and used an ingredients-based approach. All individuals enrolled in the C No More study from 21 September 2023 to 31 March 2025 were accounted for, and the analysis covered all costs from initial testing to posttreatment sustained virological response (SVR) testing (where applicable) and follow-up attempts. Costs are presented in 2024 Australian dollars without discounting.

Infrastructure costs included van depreciation (based on purchase price and straight line depreciation across 18 months of 60% use (3 days per week for C No More, 2 days per week for other purposes) and an assumed 10 year lifespan; modifications made to the van for clinical needs (eg, venipuncture chair, clinical bench, and storage); the Cepheid GeneXpert testing system (point-of-care machine and laptop, provided to the study by the Australian HCV Point-of-Care Testing Program but costed for generalizability). The GeneXpert system requires a portable battery to operate in the van, as well as annual servicing and a quality assurance program, the costs of which were also included.

Clinic costs included staff salaries (1 nurse and 2 peer workers, calculated using hourly rates) and travel costs from the hospital to each clinic location. The nurse wage was determined from the Nurses and Midwives (Victorian Public Sector) (Single Interest Employers) Enterprise Agreement 2020–2024 [18], and travel costs included fuel and toll road costs.

Testing costs were broken down into HCV antibody and HCV RNA testing and included the cost of the test module (HCV antibody) or cartridge (HCV RNA), both provided by the National Point-of-Care Testing Program, as well as testing consumables, such as lancets, cleansing swabs, gloves, and bandages. Testing costs were estimated per test. Incentive costs included A$40 department store vouchers and incentive packs containing toiletries and snacks, valued at A$7.50, given to all participants on initial presentation.

Treatment initiation and monitoring costs included pretreatment workup, treatment initiation and dispensation, staff follow-up time, and cure ascertainment. The cost of pretreatment workup blood-based investigations included venipuncture consumables (eg, blood collection kit and vacutainer tubes) and treatment-based costs. Treatment-based costs included nurse practitioner time spent on writing prescriptions (estimated as 10 minutes) [18], and pharmacy dispensing fees. In Australia, pretreatment investigation laboratory costs and DAA treatment costs are covered by the Medicare and Pharmaceutical Benefits Scheme and were not included in costs. Only pharmacy dispensing fees are required to be paid by the individual, which were covered by the C No More study and included as a service provider cost (either A$23.10 per dispensation or A$6.10 for individuals with a government concession card). Dispensation costs were the same for dispensations through the van via the hospital pharmacy and via local pharmacies.

Treatment costs also included staff follow-up time associated with treatment initiation, support, and monitoring, and cure ascertainment, calculated for every individual by reviewing study records. Contact associated with the individual's treatment, including unsuccessful contact attempts and liaising with other contacts, such as family or friends of the individual, social or support workers, and pharmacists, were included in follow-up costs. If individuals had multiple treatment attempts (eg, due to treatment noncompletion), follow-up events associated with subsequent treatments were included. Cure ascertainment costs included costs of an additional A$40 department store incentive voucher for SVR (test for cure) attendance and GeneXpert RNA SVR testing costs (GeneXpert cartridge and testing consumables).

### Outcomes

The primary outcomes for this analysis were the mean program cost per individual started on DAA treatment and the mean cost per individual started on treatment that achieved cure (SVR). This was calculated by dividing the program cost by the number of individuals who commenced treatment and the number who were confirmed as achieving SVR after DAA start. The mean cost per participant and per HCV RNA–positive individual was calculated by dividing the total program cost by the number of individuals enrolled and the number of HCV RNA–positive individuals.

We also evaluated the breakdown of costs for specific pathways of the model of care. This was calculated using a decision tree to map distinct patient pathways through the model of care, beginning at HCV antibody testing, to HCV RNA testing, to DAA treatment initiation. Infrastructure and clinic costs were evenly divided by all participants to calculate a baseline value for each pathway. Specific patient pathway costs were then calculated by adding the level of care provided to each pathway of the following: (1) HCV antibody testing, (2) HCV RNA testing, (3) follow-up for treatment after positive HCV RNA result, (4) dispensing of treatment, and (5) testing for SVR.

Secondary outcomes included costs per treatment initiation and cure stratified by binary variables, current IDU status (current/former), criminal justice history (yes/no), any history of mental illness diagnosis (yes/no), current self-reported homelessness (yes/no), currently prescribed opioid agonist therapy (yes/no), and self-reported prior hepatitis C positivity (yes/no). Mean costs across groups defined by each categorical variable were compared using paired 2-sample *t* tests, with results reported as *P* values. We assumed a significance level of .05.

### Ethics and Consent

This study was approved by the St Vincent's Hospital Melbourne Human Research Ethics Committee (reference 025/023).

## RESULTS

Between September 2023 and March 2025, a total of 167 C No More “clinics” were delivered, and 832 participants were enrolled. Among 832 patients, 592 were antibody tested, 35 were antibody positive, 275 were tested for HCV RNA. Of those undergoing RNA testing, 61 were HCV RNA positive, 58 started treatment (4 were treated outside of the program), 43 were tested for SVR, and 41 achieved SVR (Table 1).

**Table 1.** Numbers of Participants by Subgroup

Subgroup	Participants, No. (n = 832; 167 Clinics)
Antibody tested	592
HCV RNA tested	275
HCV RNA positive	61
Starting treatment	58
Tested for cure	43
Cured	41

Abbreviation: HCV, hepatitis C virus.

The total cost of the C No More model of care between September 2023 and March 2025 was A$312 166.85 (Table 2). The cost per participant starting treatment (n = 58) was A$5382.19, and the cost per participant cured (n = 41) was A$7613.83.

**Table 2.** Costing Estimates for C No More Model of Care

Item	Units	Unit Cost, A$^a^	Total Cost, A$^a^	Source/Notes
Infrastructure costs				
POC machine and laptop system	1	19 285.00	19 285.00	Provided by POC testing program
Portable battery	1	1279.20	1279.20	For operating GeneXpert POC testing machine
Van modifications	1	5000.00	5000.00	Clinical bench, venipuncture chair, all other modifications
Van cost	1	8000.00	8000.00	Purchase cost estimated at A$100,000, assuming a 15-y lifespan, adjusted for 3 d/wk usage for 2 y
POC operating costs	1	6440.00	6440.00	Annual servicing and quality assurance
Clinic costs				
Nurse wage	167	465.15 per clinic	77 680.05	A$62.02/h, 7.5 h per clinic
Peer worker wage	167	627.00 per clinic	104 709.00	A$41.80/h, 7.5 h per clinic, for 2 peers
Fuel and tolls	167	11.30 per clinic	1887.10	Average fuel cost, distance estimated per clinic
Incentive costs				
Incentive packs	832	7.50 per participant	6240.00	Toiletries, deodorant, snacks
Incentive vouchers	832	40.00 per participant	33 280.00	A$40.00 Coles Myer voucher per participant
Testing costs				
HCV antibody test	592	11.00 per test	6512.00	INSTI and Abbott Bioline test cost per unit, provided by POC testing program
Antibody testing consumables	592	0.50 per test	296.00	Lancet, adhesive bandage, gloves, cleansing swab, disposable underpads
GeneXpert cartridge	275	57.00 per test	15 675.00	Provided by POC testing program
HCV RNA testing consumables	275	2.00 per test	550.00	Lancet, adhesive bandage, gloves, cleansing swab, disposable underpads, Minivette collection tube
Treatment initiation and monitoring costs				
Venipuncture consumables	45	11.00 per test	495.00	Blood collection kit, vacutainer tubes
Prescription writing	9.67	70.13 per hour	677.91	Estimated total nurse practitioner time writing prescriptions (in hours)
Dispensing fees	54	16.05 per script	866.70	Total dispensing fees calculated and averaged by participant
Staff time on follow-up for treatment	61	312.08 per participant	19 036.88	Nurse time (A$62.02/h) associated with follow-up of HCV RNA–positive participants and arrangement of treatment was calculated, with a mean of 5.02 h per participant
GeneXpert cartridge (SVR testing)	43	57.00 per test	2451.00	Provided by POC testing program
SVR RNA testing consumables	43	2.00 per test	86.00	Lancet, adhesive bandage, gloves, cleansing swab, disposable underpads, Minivette collection tube
SVR incentive vouchers	43	40.00 per participant	1720.00	A$40.00 Coles Myer voucher per participant
Total costs				
Infrastructure and clinic	…	…	224 280.35	…
Testing	…	…	62 553.00	…
Treatment initiation and monitoring	…	…	25 333.50	…
Total C No More cost (Sep 2023 to Mar 2025)	…	…	312 166.85	…
Mean total costs				
Per C No More participant (Sep 2023 to Mar 2025)	…	…	375.20	…
Per HCV RNA positive diagnosis	…	…	5117.49	…
Per participant starting treatment	…	…	5382.19	…
Per participant cured	…	…	7613.83	…

Abbreviations: HCV, hepatitis C virus; POC, point-of-care; SVR, sustained virological response.

^a^Costs are given in 2024 Australian dollars.

Of the total program cost, 13% (A$40 004.20) was associated with infrastructure costs, 59% (A$184 276.15) with clinic costs, 20% (A$62 553.00) with testing and incentive costs, and 8% costs (A$25 333.50) with treatment initiation and monitoring (Table 2). Staffing costs accounted for 64% of total costs (A$201 838.50) (Supplementary Table 1).

**Figure 1.**
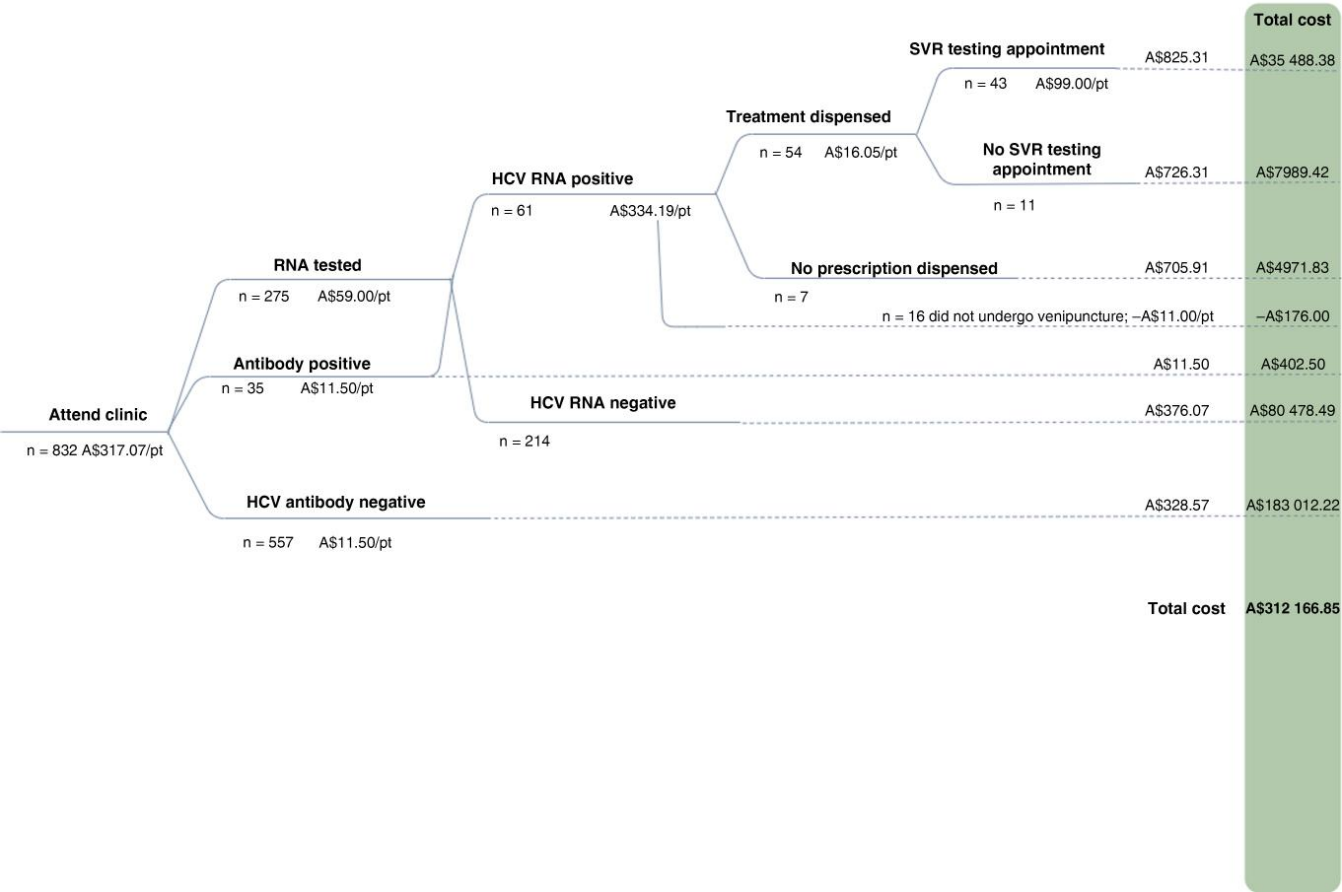
C No More patient pathway costs and total program cost (n = 832); costs are given in 2024 Australian dollars. A minority (n = 35) of individuals underwent both antibody and hepatitis C virus (HCV) RNA testing, as most individuals self-reported HCV antibody positivity and were reflexed to RNA testing. For simplicity, we have added the cost of the 35 antibody tests that preceded HCV RNA testing separately. Similarly, 16 individuals did not undergo venipuncture after testing HCV RNA positive, and we have deducted the cost from the total but did not create a new pathway. Abbreviation: pt, patient; SVR, sustained virological response.

The mean cost per participant enrolled in the study was A$375.20 (n = 832), and the mean cost per participant who tested HCV RNA positive (n = 61) was A$5117.49. Of the total program costs, 84% (A$263 490.72) was for participants who were HCV RNA negative; 14% (A$44 001.30) for participants who were HCV RNA positive and commenced treatment (n = 54), and 2% (A$4971.83) was for participants who were HCV RNA positive but did not receive treatment through the program (n = 7) (Figure 1).

**Figure 2.**
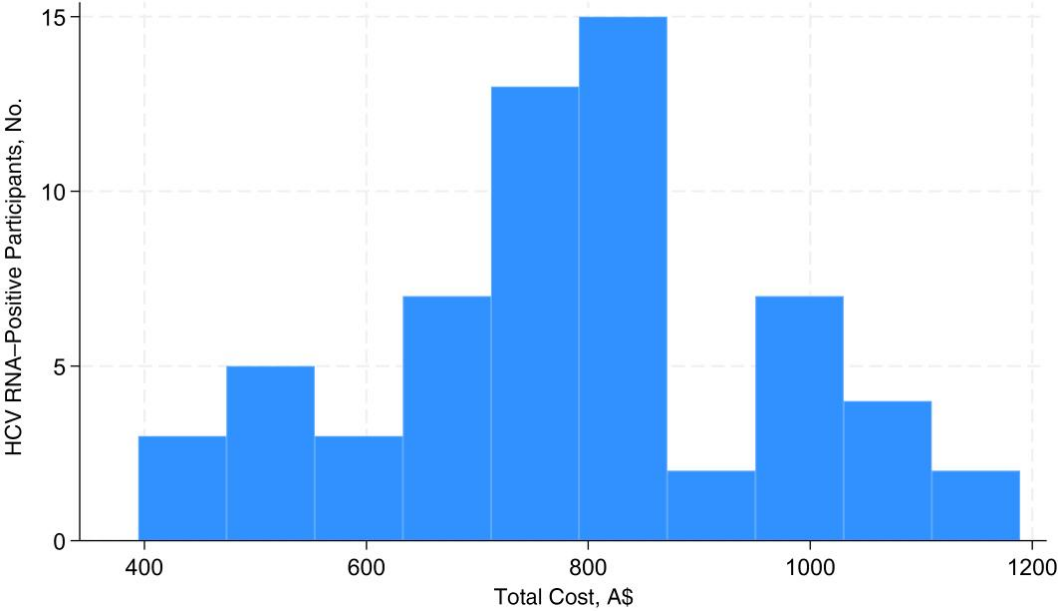
Distribution of total individual costs among hepatitis C virus (HCV) RNA–positive individuals, including costs for testing, follow-up support for treatment initiation, and sustained virological response testing (n = 61); costs are given in 2024 Australian dollars.

HCV RNA–positive participants comprised 16% of total program costs (A$48 676.13). The mean cost for HCV RNA–positive individuals was A$786.67, with a standard deviation of A$182.49 and a range from A$394.46 (uncontactable participant, did not return to clinic for follow-up or initiate treatment) to A$1188.96 (participant with no fixed address and multiple social contacts used to facilitate treatment initiation) per HCV RNA–positive participant (Figure 2).

**Figure 3.**
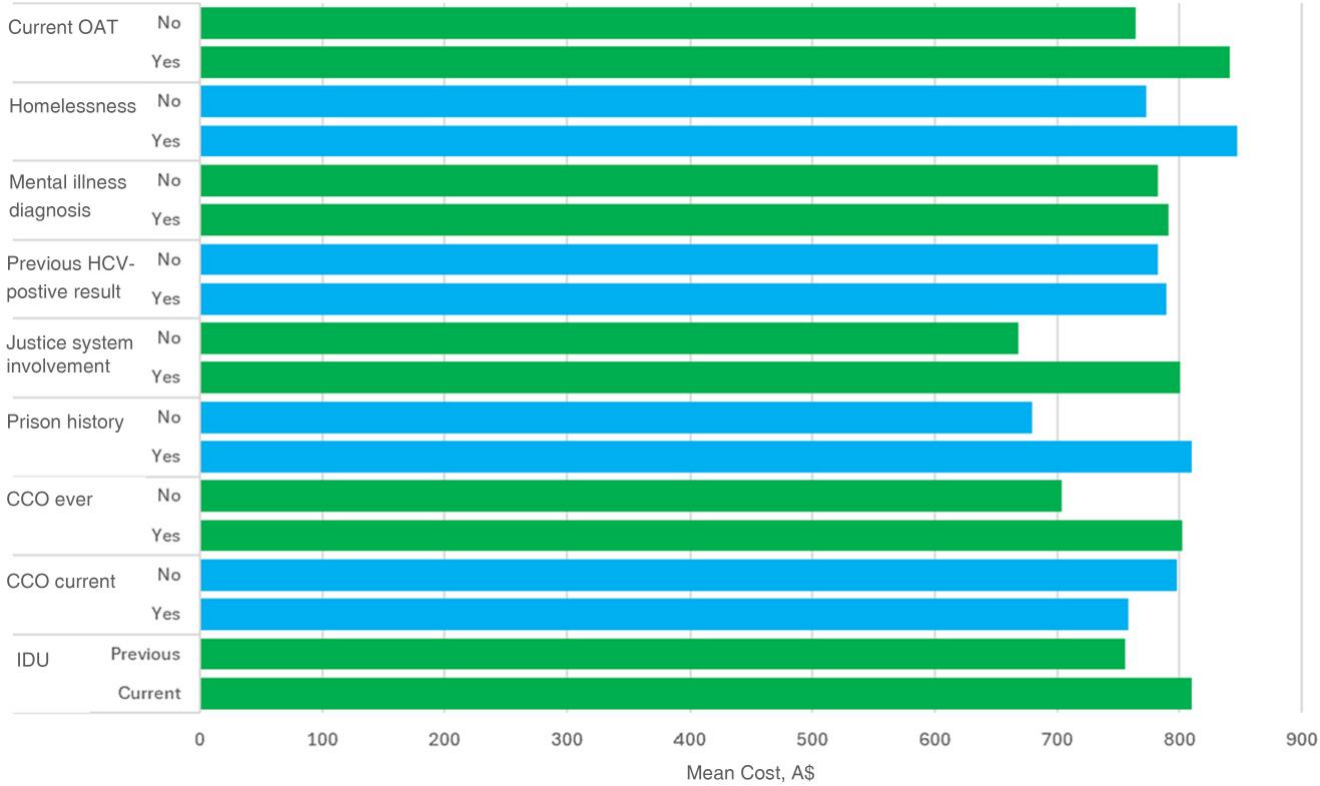
Mean total cost for hepatitis C virus (HCV) RNA–positive individuals (n = 61), by variable; costs are given in 2024 Australian dollars. Two-sample *t* tests were performed to assess mean difference within variables, and *P* values are reported in Table 3. All variables are reported for all 61 HCV RNA–positive individuals, other than injecting drug use (IDU), as 5 individuals either reported no history of IDU or declined to answer. Abbreviations: CCO, community corrections order; OAT, opioid agonist therapy.

The mean cost per HCV RNA–positive participant by categorical variable is shown in Figure 3. There was no statistically significant difference in the mean cost per HCV RNA–positive participant according to current IDU status, criminal justice history, mental illness diagnosis, homelessness, currently prescribed opioid agonist therapy, or previous hepatitis C positivity (Table 3).

**Table 3.** Mean Costs per Hepatitis C Virus RNA–Positive Participant Across Categorical Variables and Results From Independent Sample *t* Tests

Variable	Mean Cost (95% CI), A$^a^	Mean Difference (95% CI), A$^a^	*P* Value
IDU			
Current	810.17 (752.07–868.27)	55.87 (−51.16 to 162.91)	.30
Previous	754.29 (668.63–839.95)		
CCO (current)			
Yes	758.11 (666.51–849.73)	−39.59 (−144.24 to 65.05)	.45
No	797.71 (741.48–853.94)		
CCO (ever)			
Yes	802.88 (750.18–855.60)	98.88 (−25.84 to 223.59)	.12
No	704.01 (608.60–799.43)		
Prison history			
Yes	810.39 (762.50–858.27)	131.50 (13.75–249.25)	.13
No	678.89 (536.20–821.58)		
Justice system involvement			
Yes	790.66 (750.19–849.13)	132.02 (−22.52 to 286.55)	.09
No	667.64 (522.45–812.83)		
Previously HCV positive			
Yes	789.58 (734.16–844.54)	7.76 (−91.44 to 106.98)	.88
No	781.58 (688.53–874.63)		
Mental illness diagnosis			
Yes	790.66 (725.91–855.41)	8.67 (−85.91 to 103.26)	.86
No	781.98 (710.02–853.95)		
Homelessness			
Yes	847.62 (689.98–1005.27)	74.35 (−46.74 to 195.45)	.22
No	773.27 (725.27–821.26)		
Current OAT			
Yes	840.91 (777.15–904.67)	76.94 (−24.47 to 178.35)	.13
No	763.97 (703.09–824.85)		

Abbreviations: CI, confidence interval; CCO, community corrections order; HCV, hepatitis C virus; IDU, injecting drug use; OAT, opioid agonist therapy.

^a^Costs are given in 2024 Australian dollars.

## DISCUSSION

To our knowledge, the C No More model of care is the first nurse- and peer-led model of hepatitis C care to operate adjacent to the community corrections setting. Our group previously reported high levels of accessibility and acceptability from service users [19], and the current study advances these findings by describing the cost of achieving outcomes related to the primary aim of this novel model of care: to enhance uptake of hepatitis C treatment. We calculated mean program costs of A$5382.19 per treatment initiation and A$7613.83 per cure. We believe that this is the first economic evaluation of a nurse- and peer-led outreach model of hepatitis C care in Australia, and these findings could be extrapolated beyond community corrections adjacent settings to a nurse- and peer-led model of care in other settings frequented by people who use drugs, such as homelessness services.

Those delivering hepatitis C treatment in community settings need to be cognizant of the often complex needs among people most at risk of hepatitis C infection [6, 7, 17, 18]. Staff time associated with follow-up for treatment initiation and monitoring was only 8% of the total program cost, yet this supportive and person-centered follow-up is responsible for the program's high treatment initiation rates (95%) and reduced costs per treatment initiation. Our estimated cost per cure should be considered the context of providing supportive and person-centered service features designed to reduce loss to follow-up care and treatment after diagnosis. For hepatitis C treatment elimination strategies, while this adds cost, it also improves cost efficiency by reducing wasted case finding efforts (and costs) that do not result in treatment or cure and contribute to prevention of onward transmission [20]. Supportive strategies that increase retention in care are essential to ensuring that individuals receive the treatment they need and to achieving elimination goals [3].

Participants in the C No More study typically had a range of competing daily priorities that prevent engagement in traditional healthcare in the community and require resource-intensive care provision [19]. The distribution of costs associated with individuals who tested HCV RNA positive varied (from A$394.46 to A$1188.96), as some individuals had higher support needs than others. Models of care that aim to progress hepatitis C elimination goals by providing hepatitis C testing and treatment to marginalized individuals who have not yet accessed care will be more expensive, as they will require supportive, flexible systems [1]. Furthermore, we found no statistically significant difference in the mean cost per HCV RNA participant according to IDU, criminal justice history, mental illness diagnosis, homelessness, or previous hepatitis C positivity. The cost of follow-up associated with ensuring that individuals started and remained on treatment may be indicative of the cost of supportive models of care required for end-point elimination.

There are few comparable cost estimates for other hepatitis C models of care available, as many cost estimates are based on programs that restrict inclusion to individuals with current or recent IDU [21, 22] or begin with hepatitis C antibody or HCV RNA positivity and thus do not include case finding costs [23]. Furthermore, some costing estimates do not account for follow-up time [21, 22], and some programs benefit from closed environments, particularly prisons, that facilitate retention in care and do not require intensive follow-up [22, 23], reducing their comparability with our estimates.

A cost evaluation for an Australian community corrections program estimated a higher cost per cure of A$19 998 [24], despite higher HCV RNA prevalence (12% vs 7%) and similar testing rates per clinic, testing approximately 6 people per clinic (185 of 33 clinics), similar to C No More rates of 5 participants per “clinic” (832 in 167 clinics). The Queensland model is led by general practitioners and does not include follow-up costs, indicating that the C No More model is more cost efficient [25]. Nurse-led models of care and task shifting to nurses can be cost effective [23, 26, 27], reducing program costs and costs to the broader healthcare system. For example, in this model of care, a nurse practitioner assesses liver fibrosis using transient elastography (FibroScan), which may otherwise have been performed by a gastroenterologist, reducing costs to the healthcare system.

Clinic staffing costs for the nurse and peers accounted for 58% of total costs; the 2 peer workers accounted for 33% of total costs. Staffing a service like C No More requires a balance between providing an appropriately supportive and peer-led model of care and minimizing costs. For this service, a substantial peer presence is important; peer workers facilitate a comfortable, nonjudgmental environment and provide support for attending appointments and during treatment, as well as harm reduction advice. Here, the cost of peers need to be weighed against the costs associated with diagnosis in a patient who is not retained in care and treated. A key factor in the success of this model of care is the engagement and retention of people who may not have otherwise accessed or stayed in care; a mixed methods appraisal of this service found that peer workers were an important factor that increased acceptability of care [19]. Services that aim to use a nurse- and peer-led model of care will need to prioritize a system that works for both the staff and the participants and is also cost effective. Future changes to scope of practice that broaden the roles of peers may also result in lower costs associated with task shifting.

The cost per participant who started treatment (n = 58) was A$5382.19. This is considerably higher than cost per treatment initiation estimate of the nurse-led Victorian prison program (A$1802) [23] and other prison programs (A$1426) in Australia [22]. It is important to consider these estimates in the context of a dynamic, declining hepatitis C prevalence due to testing and treatment efforts. Data from the Victorian cost evaluation by Palmer et al were from 2015–2016 [23], and Shih et al used data from 2014–2019 [22]. The Australian government subsidized DAAs in 2016, and recent prison HCV RNA prevalence estimates (2022–2023; national estimate, 8%) [28] are considerably lower than earlier years in the DAA era (New South Wales in 2018, 19%) [29]. Historical Australian prison HCV RNA prevalence data are scarce [30], but a declining prevalence is also reflected in treatment numbers; in 2016 Victoria treated 454 people in prison, compared with 236 in 2023 [6, 31]. Prison-based hepatitis services provide a significant amount of hepatitis C treatment, but as hepatitis C prevalence decreases and elimination goals get closer, the cost of finding and treating hepatitis C cases will increase [1], making it difficult to compare these cost estimates with the current, late-phase elimination hepatitis C landscape.

For previously published comparable cost estimates, our cost per cure is higher than estimates of an Australian needle and syringe program (A$1248) [21, 22], which may reflect a higher prevalence of hepatitis C RNA viremia (24%) and thus higher rate of case finding and reduced cost per case found. However, their costs did not account for dispensation payments, follow-up, treatment monitoring, or testing for cure, or staff time not spent directly testing or writing prescriptions for treatment. Cost estimates from a needle and syringe program in the United States were higher ($7971 [USD] per individual starting treatment) than the C No More model [32] and also did not include staff time not spent directly testing or arranging treatment. However, Australian data indicate that HCV RNA prevalence is declining among attendees at needle and syringe programs [33], indicating that new settings may be required to reach people who still need hepatitis C treatment.

The mean cost per participant testing HCV RNA positive (n = 61) was A$5117.49. The service tested a large number of people and found a high prevalence of hepatitis C viremia (7%); higher than recent community estimates (0.5%) [34] and similar to recent Australian prison estimates (8%) [24]. That the C No More service identified a high prevalence of viremia indicates that this population is not accessing traditional healthcare services for hepatitis C care and that other strategies are required. If the people who remain to be treated for hepatitis C are highly marginalized, without access to traditional healthcare, providing hepatitis C care through integrated, and low-threshold services like the outreach, drop-in C No More service, will be crucial for achieving elimination targets [1, 35, 36].

Incentives for testing contributed to 13% of total program cost, but financially incentivizing hepatitis testing can increase the cost-effectiveness of hepatitis C models of care. Australian data indicate that incentives for engagement in hepatitis C care can increase retention and treatment initiation, and even large incentives can result in similar or improved cost per cure, particularly in settings with high rates of loss to follow-up [37, 38]. For the C No More population, the incentives were a key factor for motivating engagement in testing and many said they wouldn't have participated without the vouchers [19]. While the incentives increased the overall program cost, they were an effective engagement tool and facilitated case finding.

The majority of costs (84%) for the C No More model of care were associated with individuals who were not HCV RNA positive. To reduce total program costs, modifications to the program could be considered, such as modifying inclusion criteria to target testing to individuals with higher hepatitis C risk. However, this must be balanced with the downstream costs associated with missing diagnoses among those who either do not recall or do not disclose risk factors. Given the stigmatizing nature of disclosing drug use [35, 39], a low-threshold service that does not require disclosure of risk behaviors may be preferable. As we progress toward hepatitis C elimination and the prevalence of HCV RNA declines, some increase in case finding and overall program costs is inevitable; it is essential to capture those who are HCV RNA positive and yet to be treated but may require low-threshold care to engage.

One limitation of this analysis is the lack of similar nurse- and peer-led models of care to enable comparison of program costs. Second, we were not able to measure the outcome of treatment in nearly a third (n = 18 [31%]) of the individuals who started treatment, which affects our cost per cure estimate. The high efficacy of DAA treatment indicates that our cure rate may be higher the rate than those who have confirmed SVR, and thus our cost per cure may be an overestimate. Furthermore, our estimate of the mean cost per treatment initiation includes costs after treatment initiation including treatment monitoring and cure ascertainment (SVR testing), so our cost per treatment initiation is likely an overestimate of the cost of solely starting individuals on treatment. We use treatment initiation as a proxy for treatment completion/cure, since DAA treatment is highly effective and many individuals do not return for follow-up SVR testing.

As a third limitation, the full-time nurse practitioner role is funded by a different program, so we have accounted for only a fraction of their time spent on writing prescriptions, which is a huge concession compared with a more regular salary. Finally, our cost estimates per cure or per treatment initiation may not be directly transferrable to other community corrections office locations or other jurisdictions that may have lower HCV RNA prevalence and thus reduced cases. We estimated all follow-up occasions as an equal amount of nurse time, but there would have been considerable variation depending on individual circumstances and needs. This may underestimate the variation in cost for HCV RNA–positive individuals.

In conclusion, this is the first costing evaluation of a nurse- and peer-led model of care in Australia and in the community corrections setting, and it is reflective of a low-threshold, supportive model of care.

## Supplementary Material

ofag135_Supplementary_Data

## Supplementary Data

Supplementary materials are available at *Open Forum Infectious Diseases* online. Consisting of data provided by the authors to benefit the reader, the posted materials are not copyedited and are the sole responsibility of the authors, so questions or comments should be addressed to the corresponding author.
