# Supplementary material for: A Costing Analysis of a Nurse- and Peer-Led Mobile Model of Hepatitis C Care Adjacent to the Community Corrections Setting
Source: Open Forum Infect Dis. 2026 Mar 17;13(3):ofag135. doi: 10.1093/ofid/ofag135 (PMC13014468; doi:10.1093/ofid/ofag135)
Supplement: ofag135_Supplementary_Data [file ofag135_supplementary_data.docx]

|  |  |  |  |  |
| --- | --- | --- | --- | --- |
| **Costs** | **Number** | **Rate** | **Total cost** | **Notes** |
| **Start-up costs** | | | | |
| **Equipment** |  |  |  |  |
| POC machine and laptop system | 1 | 19285 | $19,285.00 | On-loan cost for 18-months via National Point-of-care Testing Program. Outright costs if purchased outside of the program would be approximately $64,000. |
| Portable battery | 1 | 1279.20 | $1,279.20 |  |
| Van modifications (clinical bench, venepuncture chair etc) | 1 | 5000.00 | $5,000.00 |  |
| Van cost | 1 | 8000.00 | $8,000.00 | Adjusted purchase cost for percentage of usage and lifespan. Initial estimated purchase, including to equip for clinical operations, $100,000. |
| **Operational costs** | | | | |
| **Labour** | | | | |
| **Per clinic** |  |  |  |  |
| Nurse hourly wage | 167 | 62.02 | $77,680.05 | Nurse wage * 7.5 * no. clinics |
| Peer worker wage | 167 | 41.80 | $104,709.00 | Peer wage *7.5* 2 * no. clinics |
| **Per HCV RNA positive participant** |  |  |  |  |
| Follow-up for treatment | 61 | 307.73 | $18,771.53 | Mean 10 minutes per follow-up event, mean cost of $307.73 |
| **Per participant initiating treatment** |  |  |  |  |
| Nurse practitioner time- prescription writing | 58 | 70.13 | $677.92 | NP wage*mean time (10 mins)*participants prescribed treatment |
| **Material** | | | | |
| **Per clinic** |  |  |  |  |
| Fuel | 167 | 11.30 | $1,887.10 | Average fuel cost 2023-2024/l/km, average kms across 4 clinics * no. clinics |
| **Per participant** |  |  |  |  |
| Incentive packs | 832 | 7.50 | $6,240.00 |  |
| Incentive vouchers | 832 | 40.00 | $33,280.00 |  |
| **Per HCV antibody tested** |  |  |  |  |
| HCV antibody test | 592 | 11.00 | $6,512.00 |  |
| Antibody testing consumables | 592 | 0.50 | $296.00 |  |
| **Per HCV RNA tested** |  |  |  |  |
| GeneXpert cartridge | 275 | 57.00 | 15675 |  |
| RNA testing consumables | 275 | 2.00 | $550.00 |  |
| **Per HCV RNA positive participant who undertook venepuncture** |  |  |  |  |
| Venepuncture consumables (blood collection kit, vacutainers) | 45 | 11.00 | $495.00 |  |
| **Per participant who initiated treatment who returned for SVR testing** |  |  |  |  |
| GeneXpert cartridge | 40 | 57.00 | $2,280.00 |  |
| RNA testing consumables | 40 | 2.00 | $80.00 |  |
| SVR incentive vouchers | 40 | 40.00 | $1,600.00 |  |
| **Overhead** | | | | |
| Point-of-care machine annual servicing and quality assurance | 1 | 6440.00 | $6,440.00 | Includes initial staff training. Cost of annual servicing and quality assurance outside of the National Point-of-care Testing Program may be different. |
| **Per participant who had treatment dispensed** |  |  |  |  |
| Dispensing fees | 54 | 16.05 | $866.70 |  |
